# *Cheiracanthium ilicis* sp. n. (Araneae, Eutichuridae), a novel spider species associated with Holm Oaks (*Quercus ilex*)

**DOI:** 10.3897/zookeys.601.8241

**Published:** 2016-06-29

**Authors:** Eduardo Morano, Raul Bonal

**Affiliations:** 1 DITEG Research Group, University of Castilla-La Mancha, Toledo, Spain; 2 Forest Research Group, INDEHESA, University of Extremadura, Plasencia, Spain; 3 CREAF, Cerdanyola del Vallès, 08193 Catalonia, Spain

**Keywords:** Cheiracanthium ilicis sp. n., DNA taxonomy, Iberian Peninsula, isolated trees, molecular phylogeny

## Abstract

We describe a novel species *Cheiracanthium ilicis* **sp. n.** (Araneae, Eutichuridae) collected in the province of Toledo (Central Spain). It was found during a systematic sampling campaign carried out in an agricultural landscape with isolated Holm oaks *Quercus ilex* and small forest patches. Its morphology and affinities with other species of the genus are discussed. Furthermore, one mitochondrial gene was sequenced to confirm species membership and its differentiation from other *Cheiracanthium* species. The molecular phylogenies based on mitochondrial and nuclear genes showed a close relationship of *Cheiracanthium ilicis* **sp. n.** with *Cheiracanthium inclusum* and *Cheiracanthium mildei*, with which it also shares morphological similarities. Nonetheless, the sparse sampling of the phylogeny, due to the low number of sequences available, impedes drawing any definitive conclusion about these relationships; it is first necessary to perform an extensive review of the genus worldwide and more thorough phylogenies. *Cheiracanthium ilicis* **sp. n.** also shares certain ecological and phenological characteristics with *Cheiracanthium inclusum* and *Cheiracanthium mildei*. Like them, *Cheiracanthium ilicis* **sp. n.** is an obligate tree dweller that prefers a tree canopy habitat and reproduces primarily in late spring and summer. From a conservation perspective, the present study suggests the need to preserve isolated trees in agricultural landscapes. They are not only the refuge of common forest organisms but also of novel species yet to be discovered.

## Introduction

*Cheiracanthium* C. L. Koch, 1939 is the only genus of the family Eutichuridae Lehtinen, 1967 in Europe. This genus was transferred from the family Clubionidae Wagner, 1887 to Miturgidae Simon, 1886 (Ramírez et al. 1997) and, more recently, to the family Eutichuridae Lehtinen, 1967 (Ramírez 2014). The debate on the taxonomical status of this genus of spiders still remains open, since Wunderlich (2012) revalidated the genus *Chiracanthops* Mello-Leitão, 1942, which would include some of the species currently ascribed to *Cheiracanthium*. The spiders of this latter genus would be characterized by certain structures of the external sexual organs: male pedipalp apophysis and female copulatory ducts (Wunderlich, 2012).

*Cheiracanthium* has worldwide distribution and is only absent from the polar regions. Of the 209 known species of this genus in the world (World Spider Catalog 2016), 29 have been found in Europe, 14 of which on the Iberian Peninsula (Morano et al. 2014). With regard to the Iberian Peninsula, Urones (1987) provided the first extensive report on this genus including data on taxonomy, biology, habitat and geographical distribution of the 12 species cited up to that time. Later, Piñol et al. (2010) and Wunderlich (2012) increased this list by adding two new species observed in the Ibero-Balearic region.

Spiders of this genus are swift hunters on woody or herbaceous plants, and their dense claw tufts help them to crawl along inclined surfaces. Their body colours usually range from yellow to greenish, with orange and brownish tones in some species. *Cheiracanthium* spiders belong to the group known as “sac spiders” because they spin small silk bags which shelter these nocturnal hunters during the day. These bags are quite conspicuous, as often the spiders build them on top of tall grass shoots and so they are easily seen in wet meadows, on crops and besides paths.

In this article we describe a novel species of *Cheiracanthium* found during a sampling campaign carried out in Central Spain. Different habitats were periodically sampled in an agricultural landscape with isolated oaks and forest fragments interspersed between crop fields and grasslands. In addition to its morphological description we sequenced two genes (mitochondrial and nuclear) to build a molecular phylogeny and assess its status with respect to those species of the genus for which molecular data were available in GenBank. Finally, we provide data on habitat selection and phenology recorded during a one-year long systematic sampling.

## Material and methods

### Study area

We carried out the spider sampling in the village of Huecas, in the province of Toledo, Central Spain (40.02°N, 4.22°W; altitude 581 m.a.s.l.). The climate is dry Mediterranean, with hot summers in which temperatures may reach 40°C and scarce precipitation (365 l/m^2^) concentrated in spring and autumn. The study area extends over 9 km^2^ of flat agricultural landscape with isolated Holm oaks *Quercus ilex* and forest plots interspersed within a matrix of grasslands and cereal fields (Picture 1; see Bonal et al. 2012 for a detailed description). Tree density in the forest plots ranges from 20 to 50 trees per ha, whereas the distance between isolated oaks ranges from 40 metres to more than two kilometres.

**Picture 1.**
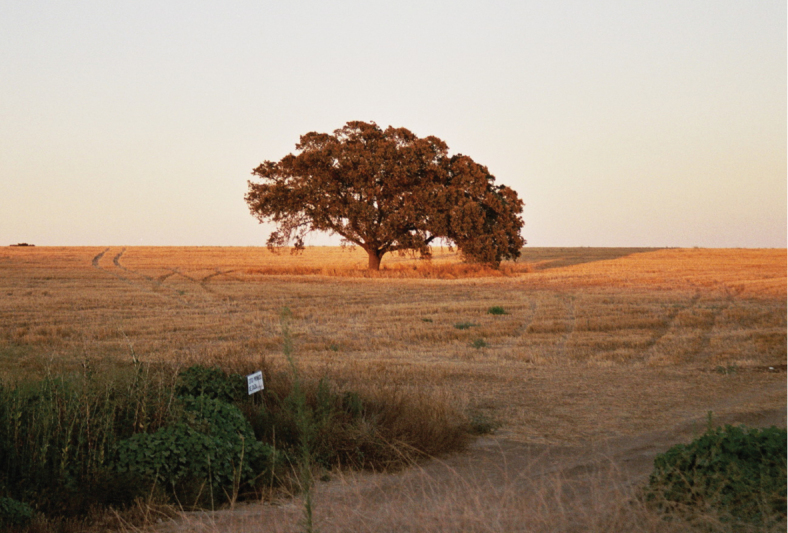
Isolated Holm oak *Quercus ilex* in the study area.

### Sampling procedure

In 2013 we conducted a systematic sampling, collecting spiders once a month from January to December. We sampled four different habitats: tree branches, tree trunks, grasses and soil. We then randomly selected 23 Holm oaks (isolated ones and within a forest patch). Tree spiders were collected by shaking the branches, and beating the canopy of each tree six times in each cardinal direction. We placed a white sheet below the branches and immediately collected all of the spiders falling onto it. Trunk traps consisted of a mosquito net attached to the tree trunk with an inverted cone with a closed bottle on the top. They covered the trunks partially and trapped the spiders that climbed the trunks and eventually walked into the net. At 10–15 meters from each study tree we set up a pair pit-fall traps separated by 10 metres. These traps were located in grasslands and consisted of a cone through which ground-dwelling spiders fell into a bottle filled with 90% ethanol and 10% glycerine to preserve the specimens. The traps were protected from direct sunlight by a small plastic roof to prevent alcohol evaporation. Lastly, grass spiders were sampled using a sweeping net along two 10-m long transects on both sides of the straight line joining the two pitfall traps. All specimens were preserved in 96% alcohol for further anatomical and molecular analyses and placed individually in Eppendorf tubes with all the information on the collection date and habitat.

### Taxonomical analyses

The spiders were inspected under a Meiji EMZ-5 estereomicroscope. Drawings were made and photos were taken of specimens and their copulatory organs using a Canon EOS 350D camera connected to the estereomicroscope. All of the specimens were separated by age (adults and immatures) and sexed whenever the development of the sexual organs so permitted. In some females, the epigyne was removed, cleaned and mounted on slides for further analysis of the internal anatomy. In the case of males, only one palp was extracted for a detailed study. The epigyne and palp removed were placed in microvials within the Eppendorf tubes of the corresponding specimens. The individuals were deposited in the collection of the Museo Nacional de Ciencias Naturales (National Museum of Natural Sciences) (CSIC), Madrid, Spain (MNCN collection of non-insect arthropods; EMH collection Eduardo Morano Hernández).

In the present study the opisthosoma length has been measured without the spinnerets and the pedicel. The total leg length (femur, patella, tibia, metatarsus, tarsus) and the leg spination pattern follow the model of Davies (1994). The spination of the legs maintains the femur, patella, tibia, metatarsus, tarsus order. First, all the spines of the prolateral surface of each segment are counted, then the dorsal ones, the retrolaterals and, lastly, the ventral spines. Hence, the resulting number is usually a four-digit one. If the spination model differs between the right and left segments the number of spines in the right one is shown within brackets. All the measurements are given in millimetres.

Abbreviations: Eyes: ALE – Anterior lateral eye(s). AME – Anterior median eye(s). PLE – Posterior lateral eye(s). PME – Posterior median eye(s). imm – immatures. CS – cymbial spur; C – conductor; TA – tegular apophysis.

### Molecular analyses

To confirm the species identity of the specimens classified as *Cheiracanthium ilicis* sp. n. the DNA of three individuals was extracted following the salt extraction protocol (Aljanabi and Martínez 1997). For each individual we amplified a fragment (627 bp long) of the mitochondrial gene cytochrome oxidase I (cox1) using the universal primers pair LCOI1490 / HCOI2198 commonly used in DNA barcoding (Folmer et al. 1994). Sequence chromatograms were assembled and edited using Sequencher 4.6 (Gene Codes Corp., Ann Arbor, MI, USA). These sequences were pooled with the cox1 sequences of *Cheiracanthium* identified to the species level available in GenBank. (Accession codes JN817218.1, JN817219.1, JN018131.1, KP975945.1, KP657470.1). We compared the intra-specific genetic divergence among the three specimens of *Cheiracanthium ilicis* sp. n., and of this species with the rest of *Cheiracanthium* cox1 sequences downloaded from GenBank. Genetic divergence was calculated by dividing the number of different nucleotides by the total number of compared nucleotides (uncorrected genetic distance).

To further assess the phylogenetic relationships between *Cheiracanthium ilicis* sp. n. and the rest of the species of the genus, we concatenated the mtDNA matrix (cox1) with sequences of the 28SrRNA nuclear ribosomal gene available in GenBank for specimens identified to the species level (four spp.) (Accession codes JN817007.1, JN817008.1, JN018345.1, KM225049.1). We obtained the 28S sequenced of one individual of *Cheiracanthium ilicis* sp.n. using the primer pair (28S a: GACCTGCCTTGAAACACGGA; 28S b: TCGGAAGGAACCAGCTTACTA) (Whiting et al. 1997). The 28S matrix, including the GenBank sequences and the new sequence of *Cheiracanthium ilicis* sp.n. (GenBank accession code KX272625), was aligned using MUSCLE (Edgard 2004). The aligned 28SrRNA data matrix was combined with the cox1 for a final concatenated data matrix 915 bp long.

Before concatenating the two genes for the phylogenetic reconstruction, two gene trees (one for cox1 and another for 28SrRNA) were built to assess any significant incongruence that could prevent concatenation. In all cases (combined phylogeny and separate gene trees) Bayesian inference analyses were used as implemented in Mr Bayes 3.2 software (Ronquist et al. 2012). The nucleotide substitution models needed for the Bayesian analyses were calculated for each gene using jModelTest 0.1.1 (Posada 2008).

Both in the combined phylogeny and in the gene trees, the sequences downloaded from Genbank of the closely related genus *Clubiona lena* were included as outgroup. The parameters in all Bayesian inference analyses were set up to two parallel runs of 2 million generations each conducted using one cold and two incrementally heated Markov chains (L=0.2), sampling every 1,000 steps. The standard convergence diagnostics implemented in MrBayes and the average standard deviation of the split frequencies were checked to deduce that the Markov chain had reached stationarity. After 500,000 generations, the average standard deviation of the split frequencies stabilised in values close to zero (0.001) and the phylogenetic trees were summarised using the all-compatible consensus command with 25% burn-in.

### Statistical analyses

We used a Chi-square analysis to assess whether the percentage of *Cheiracanthium ilicis* sp. n. individuals captured from tree branches, trunks, grass and soil differed with respect to the proportion of the whole sample (including all species of spiders) captured at each of those habitats. The differences in habitat distribution between adults and immatures of this novel species were also calculated by means of a Chi-square test. The same type of analysis was used to test the phenological differences among life stages. To do so, we divided the year into four quarters starting in January and determined whether the number of immatures and adults differed over these periods.

We investigated whether the characteristics of the oaks had any effect on the number of individuals trapped. More specifically, we used a GLM (Generalised Linear Model, Poisson distribution, Logistic link function) in which the number of *Cheiracanthium ilicis* sp. n. individuals collected at each oak was the dependent variable and the size (canopy surface in m^2^) of the tree was the independent one. The number of individuals collected could be spatially autocorrelated (i. e. it could be more similar among trees nearby). Hence, we performed an additional partial Mantel test (using distance matrixes) to calculate the correlation between the number of *Cheiracanthium ilicis* sp. n. individuals and canopy surface while checking the effect of the distance between trees. Generalised Linear Models were carried out in R (R Development Core Team, 2012). Mantel tests were performed as implemented in the R package `ecodist´ (Goslee and Urban 2007). For the rest of statistical analyses we used Statistica 7.0 (StatSoft, Inc Tulsa, OK, USA).

## Results

A total of 6048 spiders were collected throughout the whole sampling campaign. During the examination of the specimens a novel species of *Cheiracanthium* was found. It was the only species of the genus *Cheiracanthium* found in the study area and a total of 179 individuals were collected: 162 immatures and 17 adults (six males and 11 females).

## Taxonomy

### Cheiracanthium ilicis sp. n.

Taxon classificationAnimaliaAraneaeEutichuridae

http://zoobank.org/F86C27CA-700B-4906-B66E-B68D56693D6D

#### Holotype.

The holotype is a male collected in Spain: Huecas (Toledo), 581 metres above sea level (40.029915°N, 4.226789°W) by E. Morano et al. on 27 May 2013. The specimen is deposited in the collection of Arachnids of the Museo Nacional de Ciencias Naturales
(CSIC), Madrid, Spain with the following reference MNCN 20.02/17491.

#### Paratypes.

2 males and 3 females: same data as the holotype (males: MNCN 20.02/17494 and MNCN 20.02/17496; females: MNCN 20.02/17492; MNCN 20.02/17493 and MNCN 20.02/17495).

#### Other specimens examined.

Collected in the same village as the holotype but on a different date the following specimens have been studied and deposited in Eduardo Morano’s personal collection: 30 Jan 2013, 6 imm (branches); 26 Feb 2013, 2 imm (branches); 21 Mar 2013, 15 imm (branches) and 1 imm (trunk); 26 Apr 2013, 8 imm (branches) and 3 imm (trunk); 27 May 2013, 4 ♀, 3 ♂, 1 imm (branches); 25 Jun 2013, 3 ♀ (branches) and 1 imm (grass); 19 Jul 2013, 1 ♀, 31 imm (branches) and 2 imm (grass); 23 Aug 2013, 14 imm (branches); 27 Sep 2013, 15 imm (branches) and 5 imm (trunk); 22 Oct 2013, 29 imm (branches) and 9 imm (trunk); 28 Nov 2013, 8 imm (branches) and 10 imm (trunk); 19 Dec 2013, 2 imm (branches).

#### Etymology.

Most of the individuals of this novel species were collected from Holm oaks *Quercus ilex*. The Latin name of this oak species (*ilex*) in its singular genitive form (*ilicis*) has been thus used to name this spider so as to link it to the main habitat it occupies.

#### Diagnosis.

**Figure 1.**
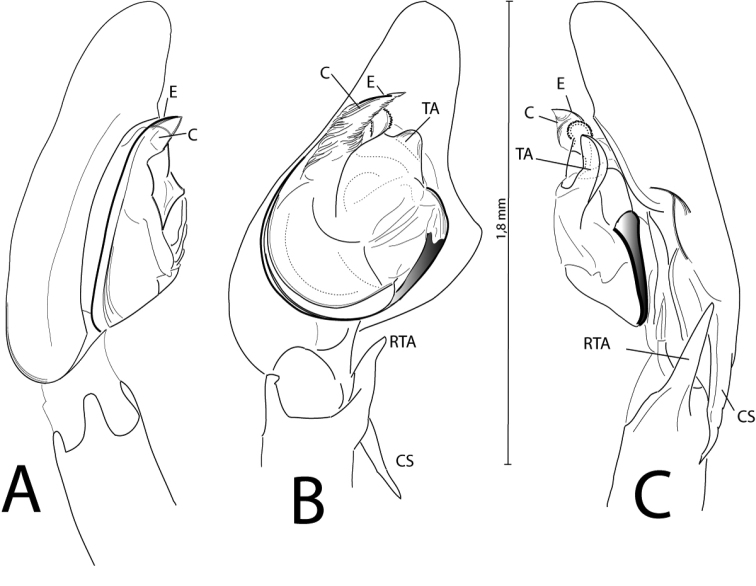
Palp of *Cheiracanthium ilicis* sp. n. In prolateral view (**A**), ventral (**B**) and retrolateral (**C**). Abbreviations: C (conductor); CS (cymbial spur); E (embolus); RTA (retrolateral tibial apophysis); TA (tegular apophysis).

**Figure 2.**
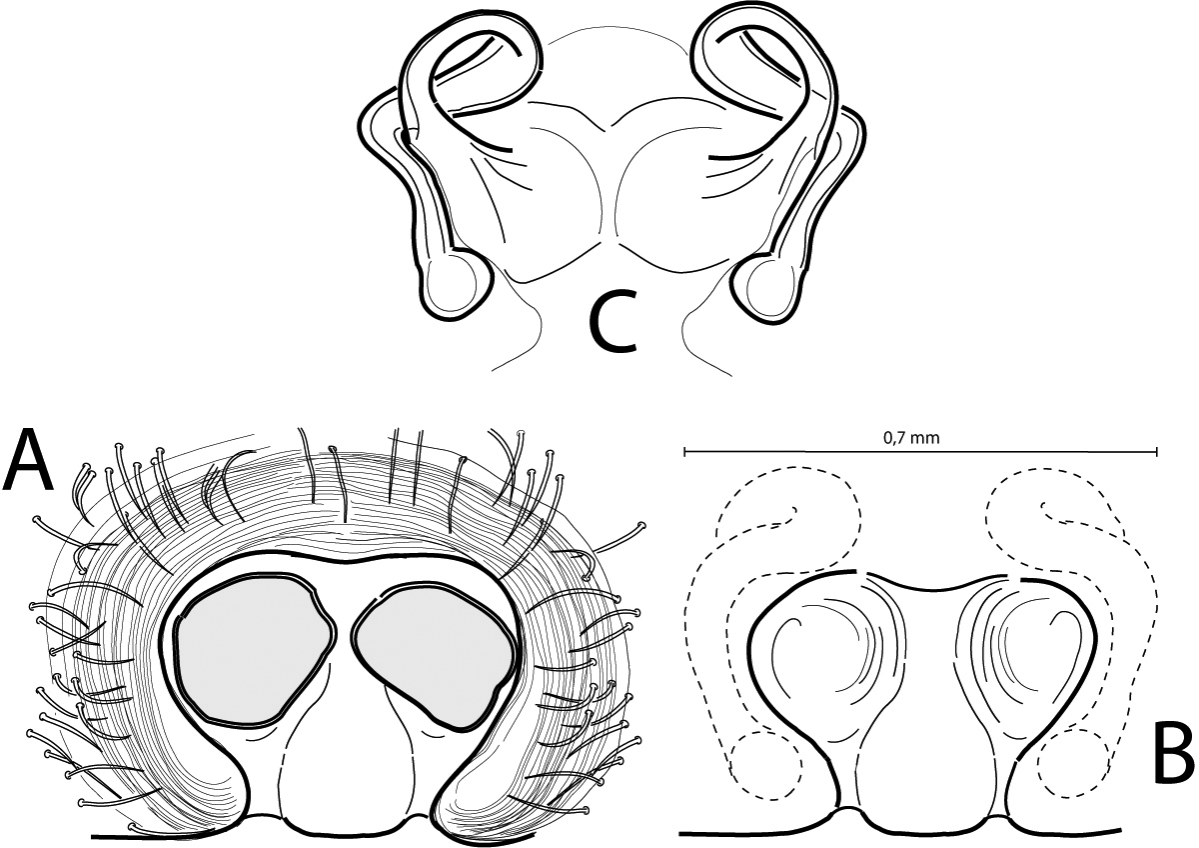
Epigyne and vulva de *Cheiracanthium ilicis* sp. n. Epigyne ventral view (**A**) with the copulatory openings closed; epigyne in ventral view (**B**) and vulva, in dorsal view (**C**).

*Cheiracanthium ilicis* sp. n. closely resembles *Cheiracanthium inclusum* and *Cheiracanthium mildei* but can be distinguished by the structure and morphology of the copulatory organs of both sexes. The palps of *Cheiracanthium ilicis* sp. n. males have a characteristic bifurcated tegular apophysis (TA; Fig. 1). Females exhibit a wide central septum that separates the copulatory openings, which are laterally opened in both depressions, and a very characteristic loop in the copulatory duct (Fig. 2).

#### Description.

Male (Holotype). All measurements are given in millimetres. Medium size. Total length: 9.4; dorsal shield lenght: 4.1; anterior dorsal shield width: 1.9; opisthosoma length: 5.0; opisthosoma width: 3.1. Eye diameter. AME: 0.150; ALE; 0.175, PME: 0.200, PLE: 0.225. Distance between eyes: AME – AME: 0.350, AME – ALE: 0.425, PME – PME: 0.225, PME – PLE: 0.275, AME – PME: 0.225, ALE – PLE: 0.05; height from clypeus to AME: 0.1; height from clypeus to ALE: 0.1.

Prosoma. Yellowish, the ocular region is darker. The immatures show a homogeneous pale green colour, changing to yellow as they reach sexual maturity (see Pictures 2 and 3). The dorsal shield has an oval shape, is slightly raised in the ocular region and has a scarcely marked thoracic furrow. The eyes are of similar size and placed in two transversal rows of four eyes each: the posterior row is longer than the anterior one; the anterior row is straight and the posterior one slightly curved; the eyes in the middle of the anterior row are less distant from each other than from the lateral ones. In the posterior row the eyes are almost regularly spaced, in such a way that the distance between the two posterior medium eyes is smaller than with those in the anterior medium eyes. The lateral eyes are very close together. The clypeus is narrower than the diameter of the AME. The labium is elongated and with a blunt end; the maxillas elongated and laterally cleaved; both structures have a light brown-orange colour with the apical ends paler, whitish. The sternum is triangular and orangish. The chelicera are dark brown-orange with a small basal condyle on its external surface. The basal segment does not have any modification and has few teeth on its margins. The promargin has two teeth, the superior one being larger; the retromargin has a decreasing series of three teeth, the first one being larger than the rest. Visualising these teeth is difficult due to the dense brush of hairs that covers them.

**Picture 2.**
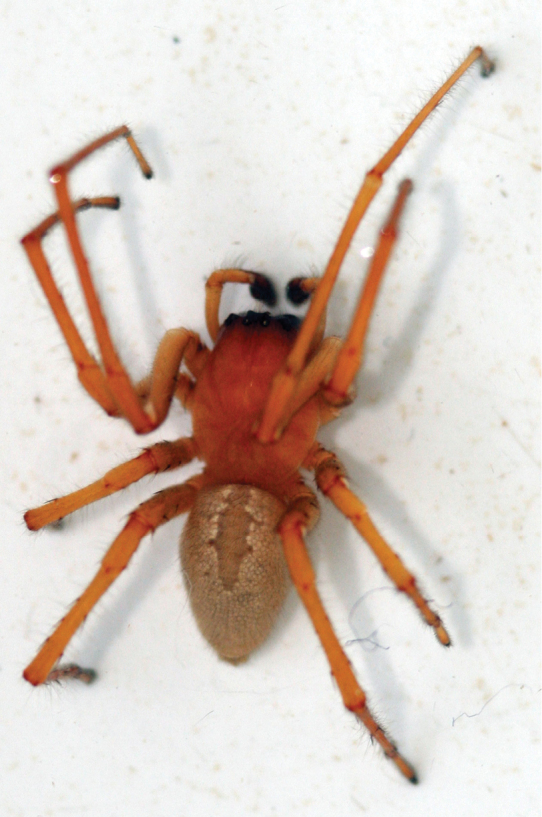
*Cheiracanthium ilicis* sp. n. adult male.

**Picture 3.**
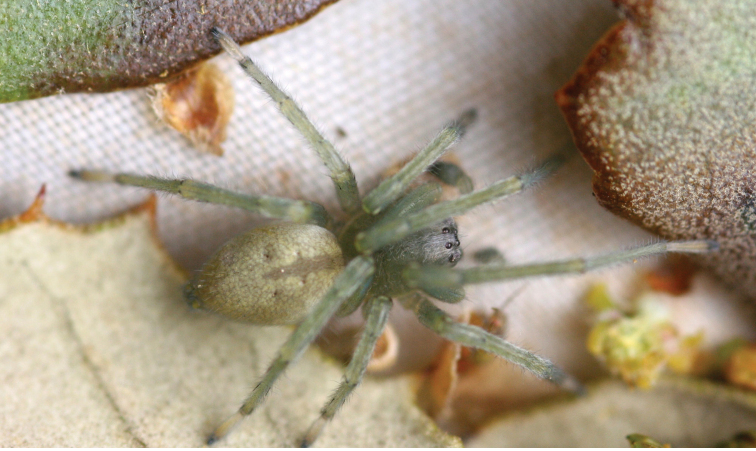
*Cheiracanthium ilicis* sp. n. immature

Legs. Yellowish and relatively long, ordered according to their length in the following way, 1:4:2:3. The trochanters show a marked external notch. The metatarsus and tarsus have scopulae on the distal portion and the latters have dense tarsal tufts.

Spination (Table 1). The palp is completely spineless as are the leg patellas and tarsus. Usually, the specimens examined have shown two pairs of femoral lateral spines and three pairs of lateral spines in the metatarsus III and IV. There is variability, for instance, in the tibia I, which can bear from nine to 11 ventral spines.

**Table 1.** Morphological measurements of *Cheiracanthium ilicis* sp. n. holotype. All measurements are given in millimetres.

holotype ♂							
leg	segment	long.	spines				
palps	Femur	1.7	0				
	Patella	0.6	0				
	Tibia	1.1	0				
	Cymbium	1.2 without or 1.8 with apophysis	0				
	total	4.6-5.2	-	leg	segments	long.	spines
I	Femur	4.8	2020	III	Femur	3.1	2020
	Patella	1.9	0		Patella	1.1	0
	Tibia	5.9	0009(00010)		Tibia	2.4	1011(1012)
	Metatarsus	5.7	0003		Metatarsus	3.2	3033
	Tarsus	1.9	0		Tarsus	1.0	0
	total	20.2	-		total	10.8	-
II	Femur	3.9	2020(2010)	IV	Femur	4.1	2020
	Patella	1.6	0		Patella	1.5	0
	Tibia	4.0	0004		Tibia	3.8	2031(3032)
	Metatarsus	4.0	0004		Metatarsus	4.7	3036
	Tarsus	1.1	0		Tarsus	1.2	0
	total	14.6	-		total	15.3	-
leg formula: I>VI>II>III							

Opisthosoma: Elongated, oval and slightly covered with pubescence, without erect antero-dorsal hairs. It has a uniform creamy colour all over its surface and its dorsum shows just a superficial heart mark, which is creamy or light green in adults and immatures respectively (see Pictures 2 and 3). The spinnerets are formed by two segments. The posterior spinnerets are longer than the contiguous anterior ones and are cone-shaped.

Male palps (Fig. 1). Most segments are light yellow with only the tarsus being brownish in colour; the tibia shows a curved retrolateral apophysis (RTA) and, in lateral view, it is inclined outwards; the tarsus is longer than the patella and the tibia taken together. The cymbium is elongated with retrolateral dilation, bearing a spur (CS) projected towards the tibia close to its retrolateral apophysis. The embolus is filiform, long and black; it starts on the retrolateral flank, surrounds the base and ends lying on the conductor (C) at the distal end of the palp. This conductor is membranous and with a pointed end. The apex hides the anterior branch of the tegular apophysis (TA). This inner branch is much more sclerotised and has the shape of a dentated disc perpendicularly oriented with respect to the external branch; the external branch of the tegular apophysis is laminar and is spoon-shaped.

Female (Paratypes, n=3). All measurements are given in millimetres. Medium size (ranges and means within brackets). Total length: 8.8–9.7 (9.13); prosoma length: 3.7–4.2 (4.00); prosoma width: 2.9–3.3 (3.06); opisthosoma length: 4.7–6.0 (5.23); opisthosoma width: 3.8–4.9 (4.30). Eyes diameter. AME: 0.200; ALE; 0.175, PME: 0.200, PLE: 0.175; distance between eyes: AME–AME: 0.300, AME–ALE: 0.325, PME–PME: 0.350, PME–PLE: 0.375, AME–PME: 0.200, ALE–PLE: 0.075, height from clypeus to AME: 0.175, height from clypeus to ALE.

In general, the appearance and colouration of males and females is similar, although the latter are larger and with shorter legs than the slender males. Compared to males, the female dorsal shield is much wider. Eye arrangement is similar in both sexes, but female eyes are a slightly larger. The margins of the chelicera bear 3 teeth (the middle one being larger) in the promargin and two teeth in the retromargin, where the first one is larger. As in the case of males, visualisation is difficult due to the dense brush of hair covering them.

Spination (Table 2). Female palp has a single, simple, straight claw. In the females examined the variability found in the spination patterns is greater than that of the males. In general, the most frequent pattern repeated has been the presence of a single spine on either side of tibias III and IV. Other than this, the spine arrangement is very variable among individuals.

**Table 2.** Morphological measurements of *Cheiracanthium ilicis* sp. n. paratypes. All measurements are given in millimetres.

		paratype ♂		paratype ♀	
legs	segment	long.	spines	long.	spines
palps	Femur	1.7–1.8	0	1.2–1.5	0
	Patella	0.5–0.6	0	0.4–0,6	0
	Tibia	1.0–1.1	0	0.8–0.9	0
	Cymbium/Tarsus	1.0–1.2 without or 1.5–1.8 with apophysis	0	1.2–1.3	0
	total	4.2–5.3	-	3.7–4.2	-
I	Femur	4.8–5.6	2020	3.8–4.3	1000(0000)-2020
	Patella	1.7–2.0	0	1.5–1.9	0
	Tibia	5.3–6.3	0009–00010(00011)	3.2–4.0	0001(0000)-0003(0002)-1001
	Metatarsus	5.3–6.5	0004–0005	3.5–4.4	0002(0001)-0005(0004)
	Tarsus	1.9–1.9	0	1.2–1.5	0
	total	19.0–22.3	-	13.2–16.1	-
II	Femur	3.7–4.1	2010(2020)-2020	2.8–3.5	1000
	Patella	1.4–1.7	0	1.0–1.5	0
	Tibia	3.4–4.3	0004(0005)-2004	2.7–2.9	0000–1000(0000)
	Metatarsus	3.7–4.7	0003(0004)-1005	2.5–3.2	0004(0003)-0005(0004)
	Tarsus	1.1–1.3	0	0.8–1.0	0
	total	13.3–16.1	-	9,9–12.1	-
III	Femur	2.8–3.2	2020	2.2–2.8	1010
	Patella	1.1–1.5	0	1.1–1.3	0
	Tibia	2.1–2.5	1010–2031(2021)	1.6–1.9	1010
	Metatarsus	2.8–3.5	3034–3035(3036)	2.0–2.3	1024(2024)-2025(2034)
	Tarsus	0.9–1.1	0	0.7–0.8	0
	total	9.7–11.8	-	7.6–9.1	-
IV	Femur	4.0–4,3	2020	3,2–3-6	1000(1010)
	Patella	1.5–1.8	0	1.3–1.7	0
	Tibia	3.2–4.0	2011(2020)-2032(2022)	2.7–3.0	1010
	Metatarsus	4.3–5.5	3035(3036)-3036(3037)	3..1–3.8	2027–2037(2038)-3027
	Tarsus	1.0–1.2	0	0.9–1.1	0
	total	14.0–16.8	-	11.3–13.1	-
leg formula		I>IV>II>III	-	I>IV>II>III	-

Epigyne (Fig. 2A, B). It is an oval-shaped plate, wider than longer, with a rim resulting from the protrusion of the spermathecae and copulatory ducts. The copulatory openings are placed laterally, protected by the rim, and in the middle zone of a spherical funnel-shaped depression. In some females these depressions were covered by a dark substance that had to be removed in order to examine the morphology of the epigyne (Fig. 2A) (Dondal and Redner 1982). In the centre of the plate a septum separates each of these two depressions. In some specimens, the transparency allows the observation of the lateral section of the copulatory ducts, and also the spermathecae located in the posterior zone of the epigynal rim (Fig. 2B).

Vulva (Fig. 2C). The copulatory ducts start in each depression and go backwards, where they are ventrally curved forming a loop and going towards the spermathecae located behind. These spermathecae are small, almost circular and separated.

##### Phylogenetic relationships

The sequence of the cythochrome oxidase I gene was identical in the three individuals of *Cheiracanthium ilicis* sp. n. (GenBank Accession code KX272624). The divergence with respect to the closest species (*Cheiracanthium mildei*) was 7.5% and 11.4% with respect to *Cheiracanthium inclusum*.

**Figure 3.**
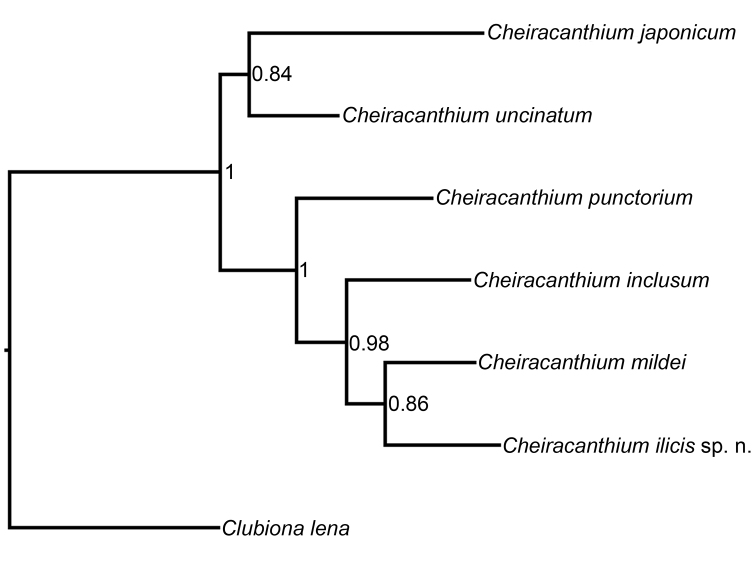
DNA phylogeny of one mitochondrial (cox1) and one nuclear (28S) genes showing the phylogenetic position of *Cheiracanthium ilicis* sp. n. within its genus. Tree topology was inferred using maximum likelihood (GTR + I + Gamma substitution model) and Bayesian inference.

The two gene trees (cox1 and 28SrRNA) showed congruent topologies, what allowed the concatenation of the sequences of both genes. Sequences of both genes were available for all species with the exception of 28SrRNA for *Cheiracanthium inclusum*. The Bayesian phylogeny combining both genes (Fig. 3) retrieved a clade with a highly supported node (PP=1) that included four species (*Cheiracanthium punctorium*, *Cheiracanthium inclusum*, *Cheiracanthium mildei* and the new *Cheiracanthium ilicis* sp. n.); within this group, *Cheiracanthium mildei* and *Cheiracanthium ilicis* sp. n. were sister groups (PP=0.86) and *Cheiracanthium inclusum* was sister to the *Cheiracanthium mildei*-*Cheiracanthium ilicis* sp. n. clade. *Cheiracanthium mildei*- *Cheiracanthium ilicis* sp. n. (Fig. 3) were also sister-species in the two gene trees.

##### Habitat distribution and phenology

**Figure 4.**
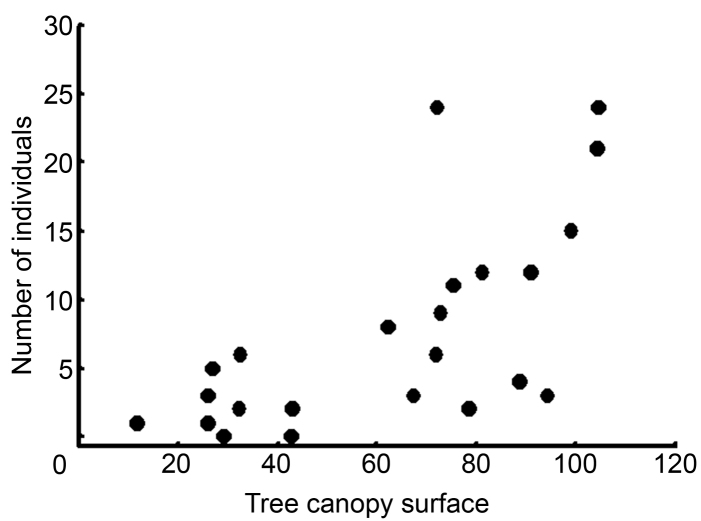
Relationship between the number of individuals collected and the tree size (canopy surface in m^2^).

The novel species of *Cheiracanthium* was not randomly distributed in the four habitats sampled (Chi=98.59; df=3; P<0.0001). Most of the individuals were collected from Holm oak branches (82.68%) and trunks (15.64%); only three (1.67%) from grass and none from pit-fall traps. All adults were captured from the tree branches but the habitat distribution differences between adults and immatures were not statistically significant (Chi=4.02; df=2; P=0.13). There was a positive relationship between the number of individuals collected from each tree and the surface of its canopy (Fig. 4; Estimate=0.026170; Z=7.894; P<0.001). The positive effects of tree size on *Cheiracanthium ilicis* sp. n. numbers were independent of the spatial distribution of the trees. The Mantel test demonstrated that the number of individuals collected was not spatially autocorrelated (R=-0.004; P=0.41) and the positive relationship between canopy surface and *Cheiracanthium ilicis* sp. n. numbers remained significant after checking the Euclidean spatial distance between trees (R=0.30; P<0.01).

**Figure 5.**
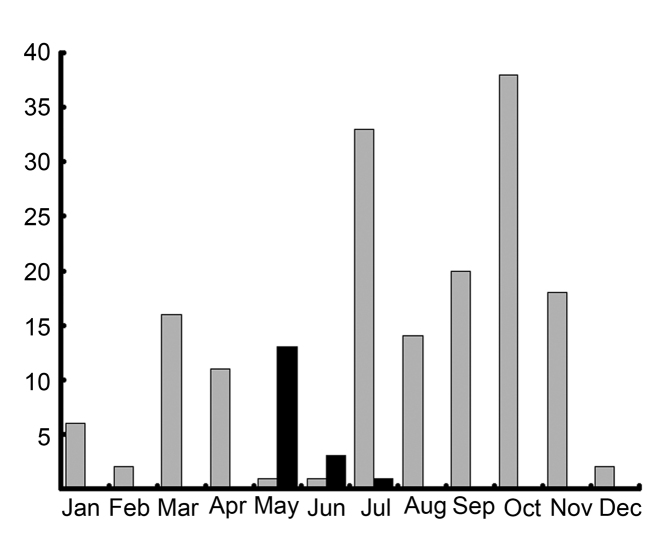
Number of immatures (grey bars) and adults (black bars) *Cheiracanthium ilicis* sp. n. collected throughout the year.

We collected individuals of *Cheiracanthium ilicis* sp. n. all year round, but the numbers were lower in the winter months (Fig. 5). There were significant differences between age classes (Chi=170.79; df=3; p<0.0001), as almost all adults were collected only in the second quarter of the year (spring-early summer).

## Discussion

We describe a novel species of *Cheiracanthium* that can be easily diagnosed based on male and female genitalia from other species in the genus. The amount of genetic divergence in DNA barcode sequence (cox1) (7 to 11 % from closest relatives) provides further support for its distinctiveness.

Morphologically, *Cheiracanthium ilicis* sp. n. resembles *Cheiracanthium inclusum* and *Cheiracanthium mildei*. Like them, *Cheiracanthium ilicis* sp. n. males have a pedipalp with a relatively stout cymbial apophysis, a strongly sclerotized, stout median apophysis and no tegular apophysis. Also, in these three species the embolus is on the retrolateral margin of the bulbus and females have relatively stout copulatory ducts that do not encircle the two pairs of receptacula seminis on the vulva. *Cheiracanthium ilicis* sp. n. shares other characteristics with these species, such as a weakly developed or absent thoracic fissure and basal cheliceral articles that, at least in *Cheiracanthium mildei*, are smaller in females and not pigmented in the distal half. Male chelicera do not have modified basal articles and are powerful and more elongated than in females, especially in *Cheiracanthium inclusum* and less in *Cheiracanthium mildei*. In *Cheiracanthium mildei* male pedipalps have an additional dorsal tibial apophysis. In turn, in *Cheiracanthium mildei* females the copulatory ducts are placed in the middle of a strongly sclerotised epigyne. The phylogenetic analysis of the concatenated cox1+28S data matrix also supports the close relationship of the new species with *Cheiracanthium mildei* and *Cheiracanthium inclusum*. The two last species are included in *Chiracanthops*, a genus recently resurrected by Wunderlich (2012), which may suggest that the new species actually belongs to the genus *Chiracanthops*. Unfortunately, the sparse sampling of our phylogeny, only six species out of the 209 known species worldwide, deters us to draw any definitive conclusion about these relationships.

The somatic traits of this species are a combination of characteristics common to several afro-tropical species–such as *Cheiracanthium aculeatum* Simon, 1884, *Cheiracanthium denisi* Caporiacco, 1939 and *Cheiracanthium furculatum* Karsch, 1879, and species with a wide geographic distribution *Cheiracanthium inclusum* (Hentz, 1847) (New World, Africa and Reunion) (Lotz 2007a; Bayer 2014; World Spider Catalog 2015). *Cheiracanthium ilicis* sp. n. presents the tegular apophysis with a configuration similar to *Cheiracanthium denisi*, although the rest of the bulb differs in the cymbial spur and the retrolateral tibial apophysis. In the case of the epigyne, it resembles those of *Cheiracanthium furculatum* and *Cheiracanthium inclusum*, yet it differs in the wide septum that separates the depressions where the copulatory openings are located and in the way these are oriented antero-dorsally in the latter; whereas in *Cheiracanthium ilicis* sp. n. it is lateral and mid-positioned. By contrast, the shape of the vulva is more similar to *Cheiracanthium furculatum* and even to *Cheiracanthium mildei* L. Koch, 1864, as in both cases the copulatory ducts form a loop analogous to that found in *Cheiracanthium ilicis* sp. n.

The new species is sympatric with *Cheiracanthium mildei*, which is native to Southern Europe (Bryant 1951) and could thus co-exist with it. The information about the distribution of *Cheiracanthium mildei* on the Iberian Peninsula is recent and scarce. It is present in the north (provinces of Guipúzcoa) (Castro 2003; Castro 2009) and on the Mediterranean coast (province of Valencia) (Barrientos et al. 2010). There is not available information for the centre and the northwest of Iberia (Morano et al. 2014). *Cheiracanthium mildei* has been found to co-exist with *Cheiracanthium inclusum* in North America, where *Cheiracanthium mildei* is an invasive species (Hogg and Daane 2011). However, *Cheiracanthium ilicis* sp. n. was the only species of the genus found in our study area.

We agree with Bayer (2014) regarding the need to perform an extensive review of the *Cheiracanthium* genus for Europe, the Mediterranean Basin, Africa and the Middle East. For example, the type material of some species like *Cheiracanthium salsicola* Simon, 1932 was insufficiently described and, unfortunately, it seems to be lost, as we could not find it even after trying to do so. There is a need to study the intraspecific variability as well as to characterise lesser known species and their affinities; the discovery of this novel species in the Iberian Peninsula supports this claim. Furthermore, it would be worthwhile to sequence the genes commonly used for DNA barcoding, such as the mitochondrial cox1, in the existing species and in all the potentially novel ones. There are DNA sequences available in Gen-Bank for very few species of this genus; only if this number increases will we be able to build more thorough phylogenies that can establish reliable phylogenetic relationships among species.

The spiders of this genus occupy different habitats and can be found in grasslands, under stones, on shrubs and trees, etc (Dondale and Redner, 1982; Urones, 1988; Lotz, 2007a; Nentwig et al. 2016). In the case of *Cheiracanthium ilicis* sp. n. it has a preference for tree canopies, where most of the individuals were collected. Closely related *Cheiracanthium inclusum* and *Cheiracanthium mildei* have also been found in woody habitats (Corrigan and Bennett 1987; Hogg and Daane 2011), in which adults reproduce. We have found silk sacks in the empty acorn cups that remained attached to the oak shoots after the acorns are dropped. Only immatures were collected on the tree trunks, where they are likely to find shelter, and just three in the grasslands, probably immatures dispersing between trees. In fact, the dispersal abilities of species like *Cheiracanthium inclusum* by excreting a long silk thread that is carried by the wind (ballooning) are well known (Peck et al. 1970). The good dispersal abilities of these spiders may also explain the lack of a spatial autocorrelation among trees in the number of spiders collected.

Based on Ysnel and Canard (1986) *Cheiracanthium ilicis* sp. n. would be a spring stenocorus species, characterized by a short biological cycle with a brief adult presence in late spring and early summer. As in the case of *Cheiracanthium inclusum* in temperate North America (Peck et al. 1970), then was when we collected adults and reproduction takes place. After that, samples are dominated by juveniles and later mostly by subadults during winter and early spring. This is because the species is probably annual like other species of the genus; adults mate in early summer and then die in winter. The number of individuals and reproducing adults collected from each tree was closely related to size (canopy surface). Foliage biomass is greater in large trees and positively correlated with the abundance of herbivore insects on which spiders prey (Halaj et al. 1998). Caterpillar samplings in our study site have indeed shown that their numbers are higher in large Holm oaks (unpublished data). Also, large trees are older and the probability of tree colonisation in fragmented landscapes increases with time (Floren et al. 2011).

*Cheiracanthium* spp. spiders are nocturnal hunters and all of the individuals that we collected during the day by branch beating were quite inactive. They are very effective predators, which probably explains their success as invaders in areas outside their distribution range (Hogg et al. 2010). These spiders feed on Lepidoptera eggs, caterpillars, leafhoppers, leafminers and other herbivorous insects, some of them being insects that feed on cultivars of economic interest (e. g. vineyards, apple tree orchards). In fact, some studies have highlighted the potential role of these spiders in pest control (Corrigan and Bennett 1987; Hogg and Daane 2011). In the case of *Cheiracanthium ilicis* sp. n. inhabiting Holm oaks *Quercus ilex*, the main tree species of man-made savannahs used for livestock rearing (so called *dehesas* and *montados* in Spain and Portugal, respectively), this role has to be taken into account. Lastly, the present study supports many others that emphasise the importance of isolated trees in agricultural landscapes (Manning et al. 2006). They not only harbour common forest organisms but are also refuges for species yet to be discovered.

## Supplementary Material

XML Treatment for Cheiracanthium ilicis
